# Shared Response Inhibition Deficits but Distinct Error Processing Capacities Between Schizophrenia and Obsessive-Compulsive Disorder Patients Revealed by Event-Related Potentials and Oscillations During a Stop Signal Task

**DOI:** 10.3389/fpsyt.2019.00853

**Published:** 2019-11-19

**Authors:** Fengqiong Yu, Xingui Chen, Lei Zhang, Tongjian Bai, Yaxiang Gao, Yi Dong, Yuejia Luo, Chunyan Zhu, Kai Wang

**Affiliations:** ^1^Department of Medical Psychology, Chaohu Clinical Medical College, Anhui Medical University, Hefei, China; ^2^Collaborative Innovation Centre of Neuropsychiatric Disorder and Mental Health, Hefei, China; ^3^Anhui Province Key Laboratory of Cognition and Neuropsychiatric Disorders, Hefei, China; ^4^Department of Neurology, the First Affiliated Hospital of Anhui Medical University, Hefei, China; ^5^Anhui Mental Health Center, Hefei, China; ^6^College of Psychology and Sociology of Shenzhen University, Shenzhen, China

**Keywords:** schizophrenia, obsessive-compulsive disorder, response inhibition, error processing, N2, P3, theta oscillation

## Abstract

**Background:** Schizophrenia (SCH) patients are at high risk for obsessive-compulsive syndrome, which can lead to difficulty in differential diagnosis between SCH and obsessive-compulsive disorder (OCD). It would be of great clinical value to identify objective markers for these diseases based on behavioral or neurological manifestations. Deficient response inhibition has been reported in both SCH and OCD; however, it is unclear if common or distinct neural abnormalities underlie this impairment.

**Methods:** To address this question, we compared Stop signal task performance and associated event-related potentials (ERPs) and event-related oscillation (ERO) among 24 SCH patients, 25 OCD patients, and 27 healthy controls (HCs).

**Results:** In successful Stop trials, both SCH and OCD patients showed prolonged Stop signal response time, reduced ERP-P3 component amplitude, and weaker theta-band synchronization compared to HCs, while there were no significant differences between patient groups. In unsuccessful Stop trials, however, SCH patients demonstrated significantly lower P3 amplitudes and weaker theta-band activity than OCD patients. In addition, Stop accuracy rate in SCH patients was negatively correlated with Positive subscale score of the Positive and Negative Syndrome Scale.

**Conclusions:** These results provide evidence that impaired response inhibition in SCH and OCD arises from common underlying neural processing abnormalities. However, the lower P3 amplitude and weaker theta-band activity in SCH patients in unsuccessful Stop trials suggest distinct neural activity patterns related to error processing. These differences in ERPs and ERO may provide clues to unique neurological abnormalities in SCH and provide objective measures for differential diagnosis.

## Introduction

Schizophrenia (SCH) patients are at high risk for comorbid obsessive-compulsive disorder (OCD); indeed, about 25% of SCH patients have clinically significant OCD (1, 2). Furthermore, OCD can occur with psychotic symptoms, and OCD patients are at elevated risk for SCH (3). This high comorbidity rate can make differential diagnosis difficult (4) despite distinct disease classifications and responses to different treatment methods (5). Thus, sensitive and objective behavioral and neurophysiological indicators for distinguishing SCH from OCD would be of great value for timely differential diagnosis and initiation of appropriate treatment.

Response inhibition is a critical ability for adaption to rapid changes in the environment as well as for social interactions. Response inhibition impairments are of clinical interest because of the potential relationship with impulsive behavior and suicide. The Stop signal task (SST) is a classic paradigm for measuring response inhibition (6). The neurological processes underlying response inhibition can be investigated by recording event-related potentials (ERPs) from the scalp, specifically ERP components N2 and P3. It has been reported that N2 and P3 elicited by the Stop signal reflect distinct cognitive process in successful and unsuccessful Stop task trials. In successful trials, N2 distributed at the frontocentral area is an indicator of conflict monitoring, while P3, which has a more central and parietal scalp distribution, reflects behavioral inhibition (7). In unsuccessful trials, N2 is related to automatic error detection. Alternatively, P3 is associated with conscious error recognition and response regulation after error commission (8). Moreover, event-related spectral perturbation analysis (ERSP) has revealed enhanced theta-band power over frontocentral sites. This event-related oscillations (EROs) is important for response inhibition because it may reflect response conflict and control processes (9, 10). The theta-band activity also shows increased synchronization in error performance and is related to action strategy adjustment (11).

Numerous studies have reported that both SCH and OCD patients showed deficient response inhibition. Hughes and colleagues found that SCH patients demonstrated slower stop-signal reaction time (SSRT) than controls (12). It was further demonstrated that the response inhibition impairment in SCH patients was associated with impaired social functioning (13, 14). Other studies have detected prolonged P3 latency and decreased theta-band oscillation strength in right inferior frontal gyrus, a key brain area for response inhibition in SCH (12, 15, 16). Impaired response inhibition has also been reported consistently in OCD as indicated by a longer SSRT (17–19). An ERP study using the SST found that OCD patients exhibited larger N2 amplitude when inhibit stop signals irrespective of symptom profile and severity (20). An fMRI study found that OCD patients showed greater activity in the left pre-supplementary motor area and reduced activity in inferior frontal gyrus during successful inhibition relative to healthy controls (HCs) (21). To sum up, both SCH and OCD patients show impaired response inhibition, but it is unclear if the neural substrates are common or distinct.

In SCH, the ERN in unsuccessful trials has been consistently shown to be blunted across a range of tasks and related to poorer executive function. The reduced ERN indicates an impairment of automatic error detection. In contrast to SCH patients, numerous studies have consistently reported ERN enhancement in OCD patients, which indicates enhanced performance monitoring (22, 23). Foti and colleagues found that reduced ERN was not specific to SCH, but was also observed in other psychotic disorders, including psychotic mood disorder and substance abuse, while Pe was blunted only in SCH but not in other psychotic disorders (24). Although numerous studies have investigated error processing in SCH and OCD patents, extant studies have not directly compared the ERN and Pe between SCH and OCD during the same task.

Using ERP measures during the SST, the present study compares the neural substrates regulating response inhibition between SCH and OCD patients. It was hypothesized that in successful Stop task trials, both patients groups showed lower N2 and P3 amplitudes as well as weaker theta-band synchronization compared to HCs. In unsuccessful Stop task trials, however, OCD patients showed enhanced N2 and P3 amplitudes compared to SCH patients and HCs because of the impaired performance monitoring in SCH. We proposed that theta-band activity were impaired in SCH and OCD compared to HC in successful stop task. But in unsuccessful task, SCH showed lower theta activity in SCH than OCD and HC.

## Methods

### Participants

Twenty-seven SCH and 26 OCD patients were recruited from out-patient and in-patient clinics at Anhui Mental Health Centre, China. All patients were diagnosed by two licensed clinical psychologists using the 10th version of the International Classification of Diseases (ICD-10). Three SCH patients and one OCD patient were removed from further analysis due to poor quality electroencephalographic (EEG) data. Of the 24 remaining SCH patients, 22 were receiving olanzapine as antipsychotic therapy, of which six were also taking selective serotonin reuptake inhibitors, and two patients were drug-naive. Of the 25 remaining OCD patients, 24 were taking selective serotonin reuptake inhibitors, one patient was also prescribed a small dose of olanzapine as an adjuvant, and one OCD patient was drug-naive. The Positive and Negative Syndrome Scale (PANSS) was used to assess positive and negative symptom severity of SCH patients. All OCD patients were assessed by the Yale-Brown Obsessive-Compulsive Scale (Y-BOCS) (25). OCD patients were also assessed using the Hamilton Anxiety Scale (HAMA) and Hamilton Depression Scale (HAMD). Twenty-seven matched HC participants were recruited *via* online advertisement. All patients were required to be normal or corrected-to-normal vision. The exclusion criteria were history of neurologic disorders, any brain injury with loss of consciousness, mental retardation or other severe developmental disorders, and history of substance abuse. The participants included in the study did not take any type of psychotherapy. All participants signed an informed consent form for the study.

### Stimuli and Experimental Procedure

**Figure 1:**
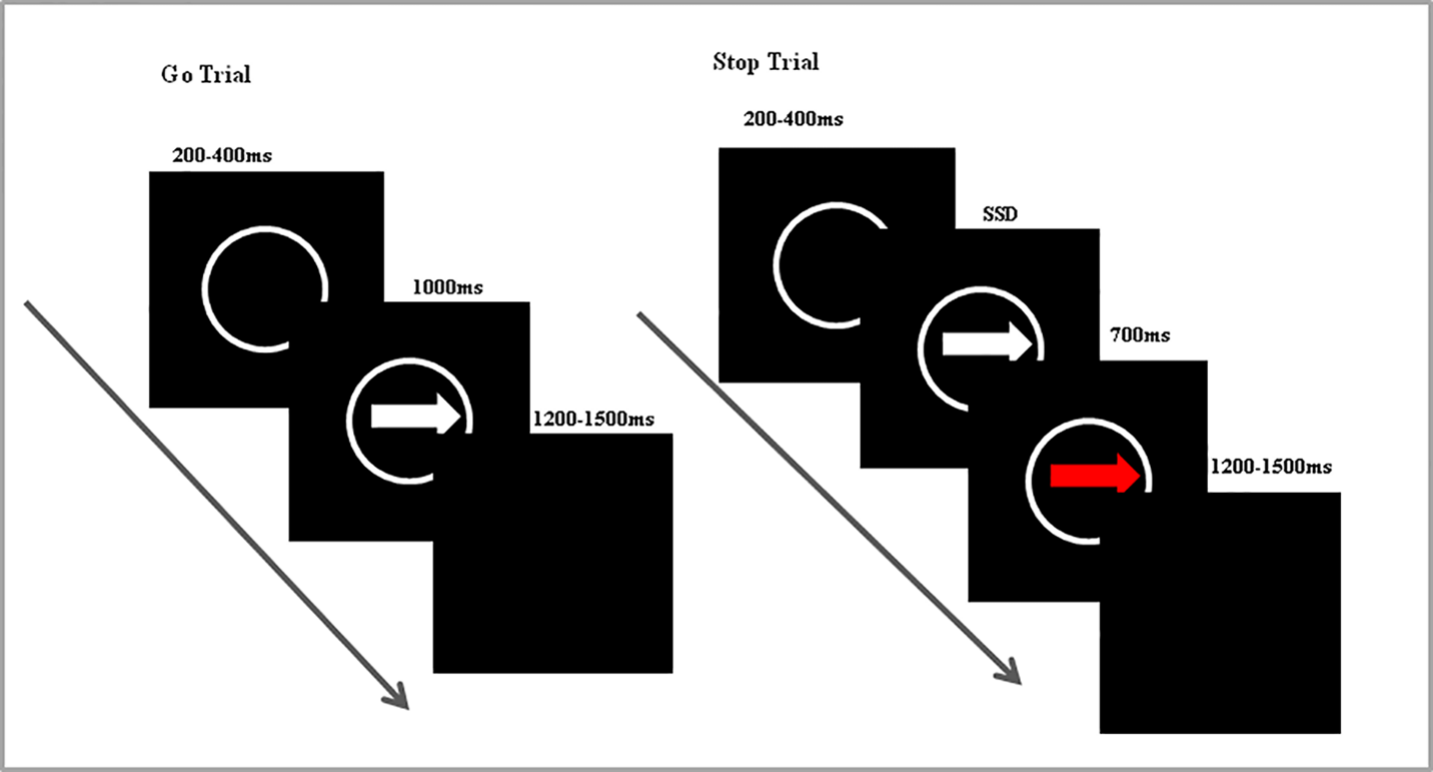
Timeline of Go and Stop trials in the stop signal task.

All study procedures were approved by the Anhui Medical University Ethics Committee and conformed to the ethical principles of the Declaration of Helsinki of 1975, as revised in 2008. This study used a modified SST with randomized design (26). Go and Stop trials were included in each session (**Figure 1**), of which 70% were Go trials. Each trial was initiated by a central white cross on a black computer screen lasting for 200–400 ms. A left- or right-pointing white arrow then appeared at the central fixation target for 1,000 ms. Participants were required to judge the orientation of the arrow as accurately and quickly as possible. The remaining 30% of trials were Stop signal trials. These trials were initially identical to the Go trials, but the arrow turned red after a variable delay (stop signal delay; SSD), cuing participants to inhibit the target response. The SSDs were dynamically adjusted using a 1-up/1-down tracking procedure, thereby ensuring successful inhibition on 50% of the Stop signal trials. The initial SSD was set at 225 ms and increased by 50 ms when the subjected succeeded in response inhibition or decreased by 50 ms when the subject failed to inhibit the response. The SSRT was estimated by subtract the mean SSD from the mean Go time (26). The inter-trial interval varied from 2200 to 2500 ms. There were three experimental blocks of 120 trials each. The entire session required about 25 minutes to complete.

### EEG Data Recording and Analysis

EEG data were recorded from 64 tin electrodes placed on the scalp according to the extended International 10/20 system using a Neuroscan recording system (Neuro Scan, Sterling, VA, USA). EEG signals were recorded using a left mastoid electrode as the online reference. All electrode impedances were maintained below 10 kΩ. EEG activities were amplified with 0.01–100 Hz band-pass filtering and continuously sampled at 500 Hz/channel.

MATLAB scripts using functions from the EEGLAB environment was adopted to process and analyze the EEG data (27). The collected data were re-referenced to the average of left and right mastoids and were down-sampled to 250 Hz. Then, the data were subjected to a high-pass filter at 1 Hz (FIR filter conducted with pop_eegnewfilt with the default parameters, cutoff frequency of 0.5 Hz, and 26 dB) to remove baseline drift, thereby ensuring reliable results for independent component analysis (28). Artifactual channels and nonbrain electrodes were rejected by the clean_rawdata plugin in EEGLAB, leaving an average of 58.52 [95%, (29, 61)] clean channels per participant. Continuous data were filtered and segmented from 1,000 ms before the go and stop signal to 2,000 ms after the stimulus. Artifactual epochs were identified and removed based on a) abnormal spectral characteristics of high frequency noise (rejspec; 20–40; <−35 or >35 dB), b) abnormal trends (rejtrend; slope > 200 µV with R2 > 0.3), c) abnormal amplitude (threshold −500 µV or +500 µV), d) improbable data using joint probability [jointprob, 8 standard deviation (SD) for single channel and 4 SD for all channels], or e) abnormal distributions (rejkurt; 8 SD for single channel and 4 SD for all channels). Data from electrodes responsible for more than 10% of rejected epochs were rejected. Subsequently, epoched data were decomposed into maximally independent components using an extended infomax algorithm implemented by the runica function with default parameters. Artifactual components electrooculogram and electromyogram were identified and removed by the EEG_SASICA plugin in EEGLAB (29). On average, there were 53.03 [95%, (27, 57)] components left per participant. The mean proportion of rejected epochs was 3.2% [95%, (1%, 9%)] in the HC group, 3.07% [95%, (0%, 9%)] in the SCH group, and 2.85% [95%, (0%, 9%)] in the OCD group. Rejection rates did not differ significantly among groups (*F*_2,82_ = 0.25, *P* = 0.78).

The time-frequency information was obtained using Morlet wavelet decomposition operated with the EEGLAB newtimef function. Spectral power was calculated with 50 log-spaced center frequencies ranging from 3 to 50 Hz, and 200 linearly spaced time bins across the epoch. Given the balance between frequency and temporal resolution, the wavelets were built on the parameter [3, 0.8] specifically for three cycles at the lowest frequency (3 Hz) and 10 cycles at the highest frequency (50 Hz). The normalized power employed a dB transform [dB power = 10*log10 (power/baseline)].

The cleaned ERP waveforms were time-locked to stimulus onset and epoched to 200 ms pre-stimulus and 1,000 ms post-stimulus. The ERPs were averaged separately for successful and unsuccessful Stop signal trials and correct Go signal trials. As N2 and P3 are common indices for response inhibition, we analyzed these two components in this experiment. N2 was defined as the peak amplitude between 150 and 250 ms and P3 as the mean amplitude within the 100-ms time window from 350 to 450 ms.

The ERSP and inter-trial coherence (ITC) measurements for successful and unsuccessful Stop signal trials and successful Go signal trials were calculated and averaged across participants. The ERSP measures changes in the amplitude of the EEG spectrum relative to experimental events. The time-frequency window of interest (TF ROI, 4–7 Hz, 200–600 ms) was selected based on evidence from previous studies and visual inspection according to the maximal strength of event-related synchronization in the theta band averaged across all subjects, and conditions.

The ERP and ERSP data were extracted from left-frontal (F3, F7, FC3, FT7), right-frontal (F4, F8, FC4, FT8), medial-frontal (Fz, FCz, Cz), left-temporal (T7, TP7), right-temporal (T8, TP8), left parietal (P3, P7, CP3), and right-parietal (P4, P8, CP4) electrode clusters according to a previous study (16).

### Statistical Analysis

All data were analyzed using IBM SPSS 16.0 (IBM Corp., Armonk, NY, USA). Chi-square tests were used to assess the difference in sex ratio between groups. One-way analysis of variance (ANOVA) was used to assess group differences in age, years of education, Go RT, SSD, SSRT, and accuracy of Go tasks (Go ACC) and Stop tasks (Stop ACC). Independent samples t-test was used to compare disease duration between SCH and OCD groups. We modeled the statistical analysis on each ERPs component and EROs power separately for successful and unsuccessful Stop task trials, as they reflect distinct cognitive process in the two tasks. In successful task, they were analyzed using repeated measures ANOVA with task (Go and SST) and clusters (left frontal, right frontal, medial frontal, left temporal, right temporal, left parietal, and right parietal) as within-subject factors and group (SCH, OCD, HC) as the between-subject factor. In unsuccessful task, they were analyzed using repeated measures ANOVA with task (Go and UST) and clusters (left frontal, right frontal, medial frontal, left temporal, right temporal, left parietal, and right parietal) as within-subject factors and group (SCH, OCD, HC) as the between-subject factor. Bivariate Pearson correlations were calculated to examine the association strengths among ERP waves and symptom severity measured by subscale of PANSS and Y-BOCS scores within SCH and OCD group. We also calculated the relationship between the symptom severity and behavioral performance (SSD, SSRT, GO RT, Stop ACC, Go ACC) of SST task within SCH and OCD group. A two-tailed *P* < 0.05 was considered significant for all tests.

The FDR (false discovery rate) method was selected for multiple comparison correction over measurements of multiple dependent variables (30). The bonferroni method was used for multiple comparison corrections in *post hoc* tests. Greenhouse-Geisser correction was used to correct p values. Partial eta squared (η_p_^2^) values were reported to examine the size of effects in the models of anova, where.05 Represented a small effect,.1 Represented a medium effect, and.2 Represented a large effect (31).

## Results

### Group Differences in Demographics and Task Performance

Demographic data are summarized in **Table 1**. There was no significant differences in age (*F*_2,75_ = 0.80, *P* = 0.46) and years of education (*F*_2,75_ = 1.02, *P* = 0.37) among the three groups. The disease duration was also not significant between SCH and OCD groups (*t*_47_ = 0.31, *P* = 0.76).

**Table 1:** Demographic data and clinical Parameters for SCH, OCD, and HC groups.

Measure	SCH(n = 24)	OCD(n = 25)	HC(n = 27)	*P* =
Age, yr	29.04(10.25)	24.70(7.93)	26.97(7.63)	F_2,75_ = 0.80/*P* = 0.46
Sex, male: female	14:10	14:11	16:11	X^2^ = 0.06/*P* = 0.97
Education, yr	12.25(2.63)	13 (3.03)	13.84(3.1)	F_2,75_ = 1.02/*P* = 0.37
Illness duration, yr	6.46(5.64)	5.93(5.03)	—	t_47_ = 0.31/P = 0.76
PASS positive	15.01(4.26)	—	—	
PASS negative	12.01(3.52)	—	—	
PASS total	55.57(9.61)	—	—	
Y-BOCS behavior	—	8.30(5.48)	—	
Y-BOCS thought	—	9.65(5.02)	—	
Y-BOCS total	—	17.97(8.43)	—	
HAMA		5.73(3.85)		
HAMD		5.89(4.33)		

HAMA, Hamilton Anxiety Scale; HAMD, Hamilton Depression Scale; HC, healthy control; OCD, obsessive-compulsive disorder; SCH, schizophrenia; Y-BOCS, Yale-Brown Obsessive-Compulsive Scale.

The three group showed significant effect on SSRT (*F*_2,75_ = 6.09, *P* = 0.01). SSRT was significantly longer in the SCH group (335.51 ± 66.05 ms) than the HC group (286.00 ± 36.36 ms, *P* = 0.016). The OCD group also exhibited marginally longer SSRT than the HC group (*P* = 0.076). The SCH group also exhibited longer SSRT than the OCD group (301.74 ± 45.45 ms), but the difference did not reach significance. In contrast, there was no significant main effect of group on SSD, goRT, goACC, or stopACC.

### Group Differences in Erps and Eros Data During Successful Stop Task Trials

#### N2 Component

The task main effect was significant on N2 amplitude (*F*_1,73_ = 19.33, *P* < 0.001, η_p_^2^ = 0.21), with significantly larger Mean N2 amplitude on successful Stop trials than Go trials (−1.36 ± 0.45 µV vs. 0.5 ± 0.18 µV). There was also a significant interaction effect between task and electrode cluster (*F*_6,438_ = 11.75, *P* < 0.001, η_p_^2^ = 0.14). The N2 amplitudes were mainly distributed at right-temporal (−3.12 ± 0.43 µV) and right-parietal areas (−4.24 ± 0.50 µV). The interaction effect between task and group was not significant (*F*_2,73_ = 1.86, *P* = 0.16, η_p_^2^^=^ 0.049).

#### P3 Component

**Figure 2:**
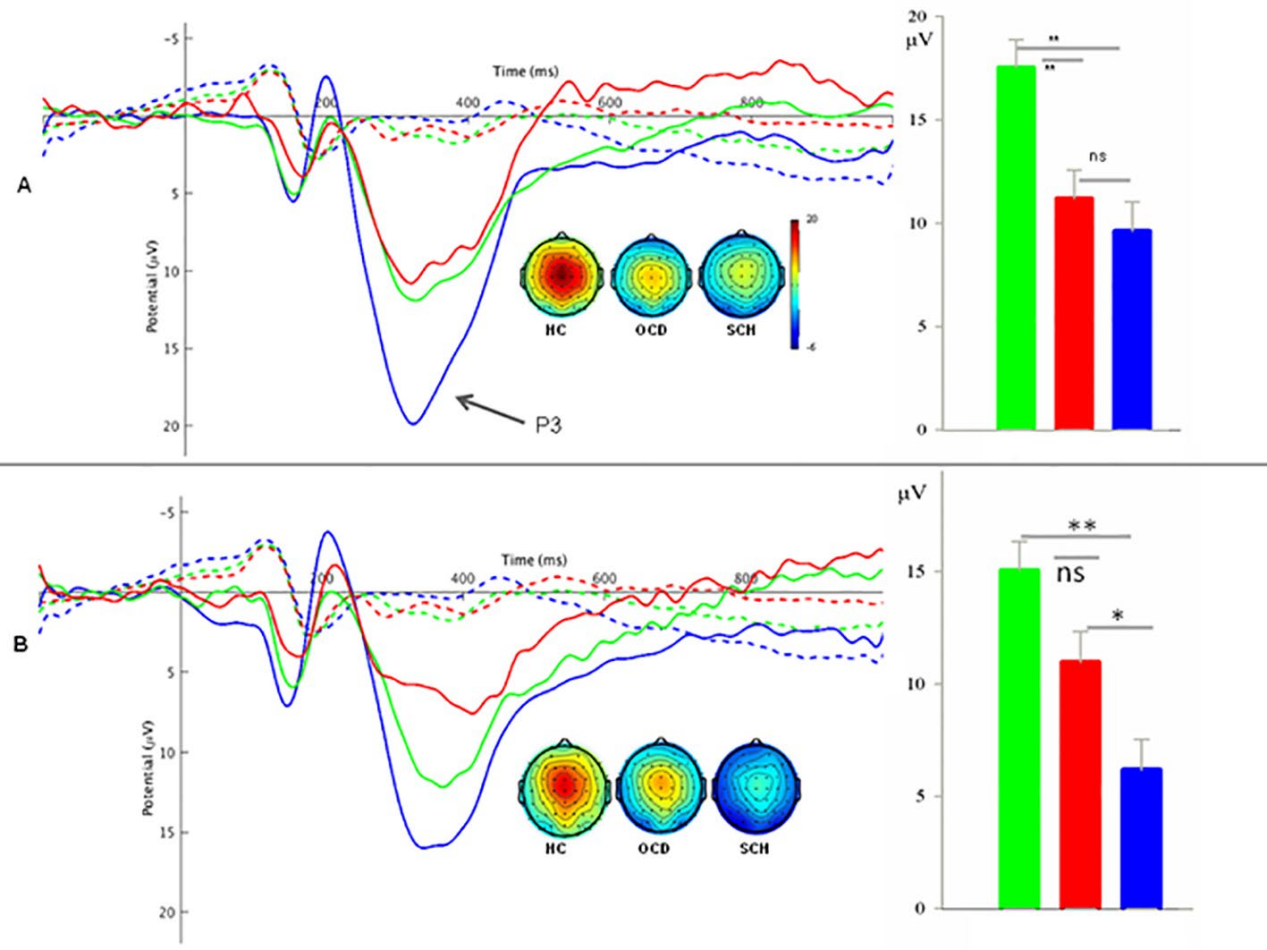
**(A)** Left panel: Grand average waves at the medial-frontal area evoked by Go signals (dashed lines) and successful Stop signals (solid lines), and the topography of the P3 component in SCH, OCD, and HC groups. Right panel: Bar graph of P3 amplitudes for the three groups in successful Stop trials. **(B)** Left panel: Grand average waves at the medial-frontal area evoked by Go signals (dashed lines) and unsuccessful Stop signals (solid lines), and the topography of the P3 component in SCH, OCD, and HC groups. Right panel: Bar graph of P3 amplitudes for the three groups in unsuccessful Stop trials. HC, healthy control; OCD, obsessive-compulsive disorder; SCH, schizophrenia. **: *P *< 0.01; *: *P *< 0.05; ns: *P *> 0.05.

The task main effect was significant on P3 amplitude (*F*_1,73_ = 140.72, *P* < 0.001, η_p_^2^ = 0.66), with greater P3 amplitude during successful Stop task trials than Go task trials (12.78 ± 0.80 µV vs. 0.83 ± 0.33 µV). Moreover, the task × group interaction was significant (*F*_2,73_ = 10.96, *P* < 0.001, η_p_^2^ = 0.23). Further analysis demonstrated a group effect in successful Stop task trials (*F*_2,__73_ = 10.41, *P* < 0.001, η_p_^2^ = 0.22) but not in Go task trials (*F*_2,73_ = 0.43, *P* = 0.65, η_p_^2^^=^ 0.012). *Post hoc* analysis revealed lower P3 amplitudes in successful Stop task trials for both OCD (11.19 ± 7.41 µV, *P* < 0.01) and SCH patients (9.61 ± 7.81 µV, *P* < 0.001) compared with HCs (17.54 ± 5.65 µV) but no significant difference was found between the OCD and SCH groups (See detail in **Figure 2**).

#### Event-Related Oscillations

**Figure 3:**
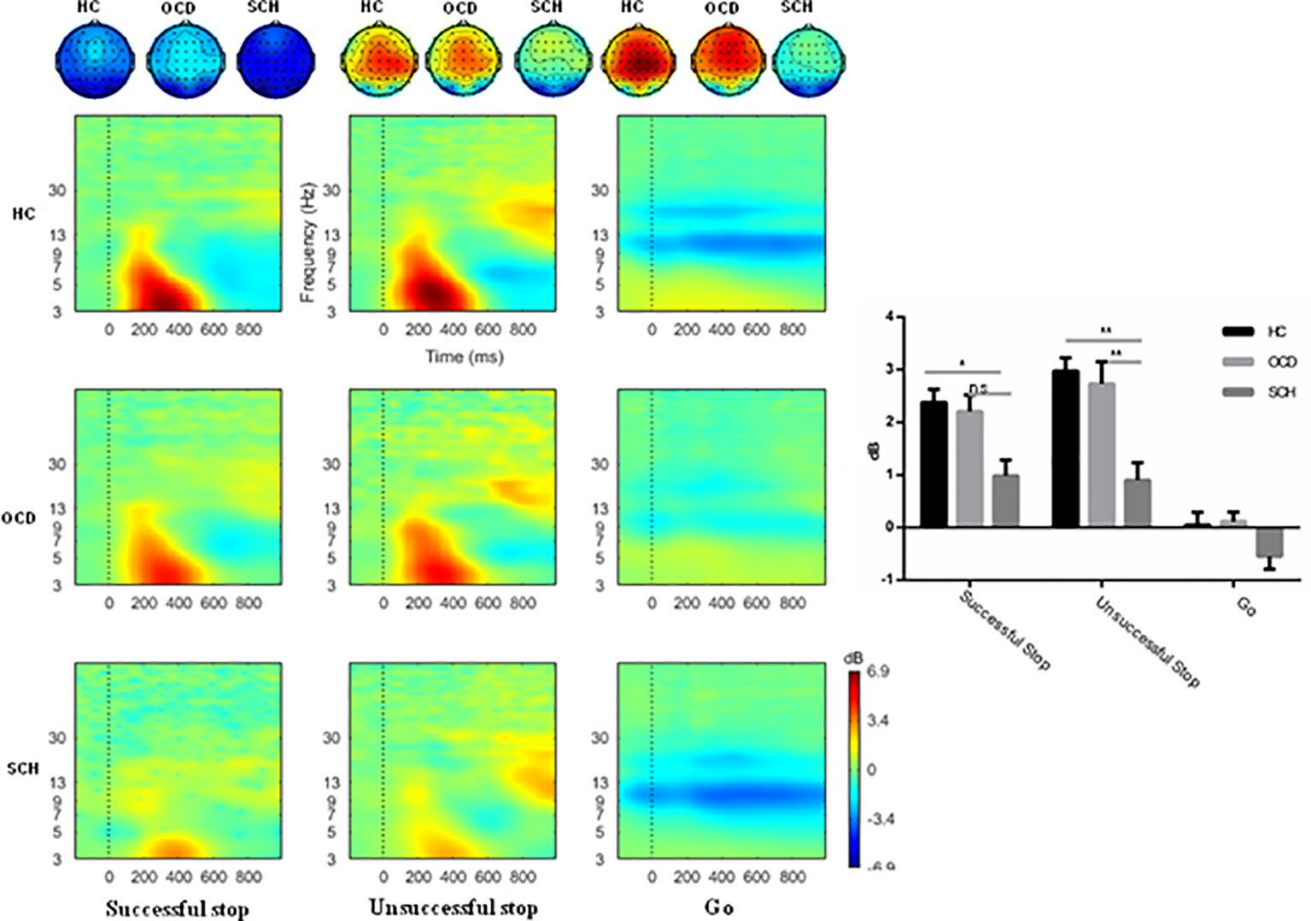
The mean ERSP image, topography, and bar graph of theta-band activity at the medial-frontal area evoked by successful Stop, unsuccessful Stop, and Go trials in SCH, OCD, and HC groups. The black rectangle defines the time-frequency window of interest. ERS, event-related spectral perturbation analysis; HC, healthy control; OCD, obsessive-compulsive disorder; SCH, schizophrenia. **: *P *< 0.01; *: *P *< 0.05; ns: *P *> 0.05.

The task main effect was significant on the spectral power of theta (*F*_1,__73_ = 112.06, *P* < 0.0001, η_p_^2^ = 0.61). Successful Stop trials elicited stronger power than Go trials. There was also a significant main effect of electrode cluster (*F*_6,438_ = 19.84, *P* < 0.0001, η_p_^2^ = 0.21), with highest power at central-frontal areas (1.07 ± 0.12 dB). Moreover, there was a significant interaction among task × cluster × group (*F*_12,__438_ = 2.34, *P* = 0.039, η_p_^2^ = 0.06), and further analysis revealed a significant task × group interaction at the middle-frontal area (*F*_2,73_ = 2.49, *P* = 0.049, η_p_^2^ = 0.064). There was a significant main effect of group on successful Stop task trials (*F*_2,73_ = 4.34, *P* = 0.017, η_p_^2^ = 0.11), and *post hoc* Bonferroni analysis demonstrated that SCH patients showed significantly reduced theta power compared with HCs (0.75 ± 0.28 vs. 1.84 ± 0.26, *P* = 0.015). The OCD patients also showed lower theta power than HCs (1.50 ± 0.26 vs. 1.84 ± 0.26), but the difference did not reach significance. The difference between OCD and SCH patients was also not significant (See detail in **Figures 3** and **5**).

**Figure 4:**
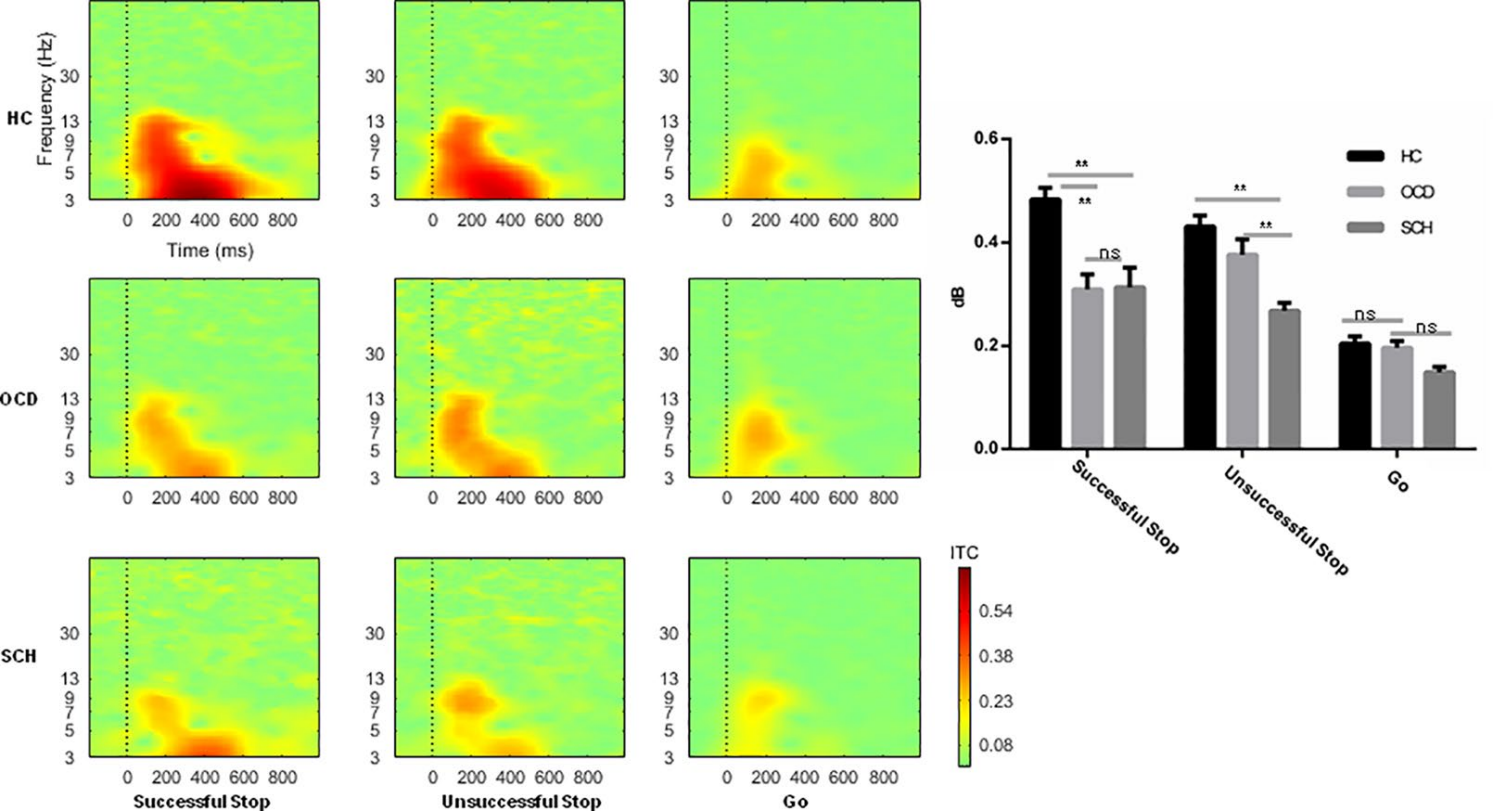
The mean ITC image, and bar graph at the medial-frontal area evoked by successful Stop, unsuccessful Stop, and Go trials in SCH, OCD, and HC groups. The black rectangle defines the time-frequency window of interest. HC, healthy control; OCD, obsessive-compulsive disorder; SCH, schizophrenia. **: *P* < 0.01; *: *P* < 0.05; ns: *P* > 0.05.

**Figure 5:**
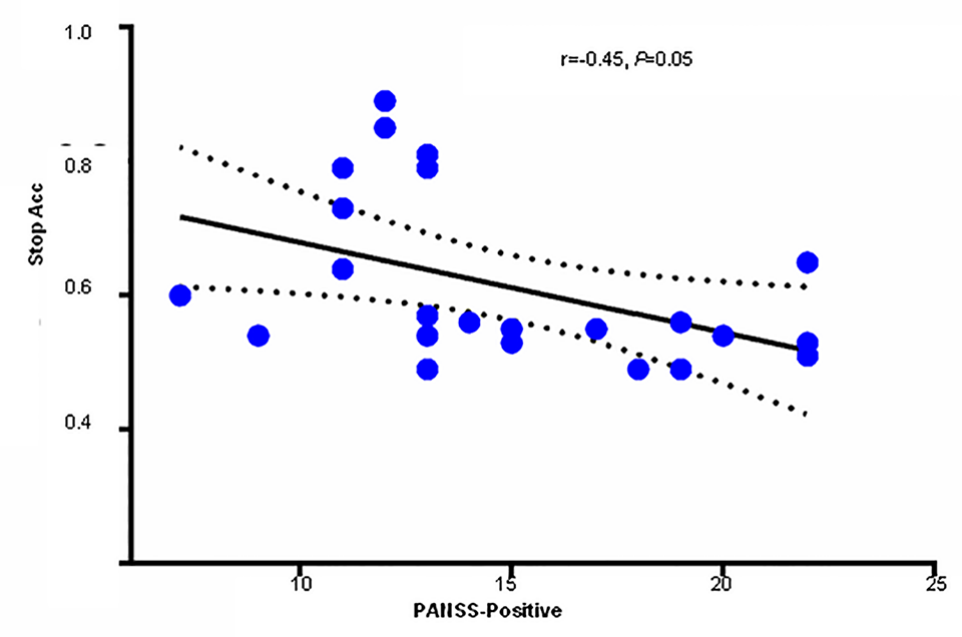
Scatter plot with Pearson’s correlation analysis demonstrating the relation between Stop ACC and positive symptom subscore on the PANSS in SCH patients. PANSS, Positive and Negative Syndrome Scale; SCH, schizophrenia.

The ITC of 4−7 Hz was analyzed during the 200−600-ms epoch. We found a significant main effect of task (*F*_1,73_ = 127.65, *P* < 0.001, η_p_^2^ = 0.64), with higher ITC in successful Stop condition trials (0.37 ± 0.016) than in Go trials (0.19 ± 0.006). More importantly, the interaction between task and group was significant (*F*_2,74_ = 10.41, *P* < 0.001, η_p_^2^ = 0.22). Further analysis showed a significant main effect of group in successful Stop trials (*F*_1,73_ = 13.23, *P* < 0.001, η_p_^2^ = 0.27). *Post hoc* Bonferroni analysis revealed significantly higher ITC in the HC group (0.48 ± 0.26) compared with the OCD group (0.31 ± 0.27, *P* < 0.001) and SCH group (0.31 ± 0.28, *P* < 0.001), but no significant difference between OCD and SCH groups (See detail in **Figures 4** and **5**).

### Group Differences in Erps and Eros Data During Unsuccessful Stop Task Trials

#### N2 Component

The task main effect was significant on N2 amplitude (*F*_1,73_ = 40.76, *P* < 0.001, η_p_^2^ = 0.36), with larger N2 amplitudes in unsuccessful Stop trials than Go trials (−1.93 ± 0.39 µV vs. 0.5 ± 0.18 µV). The interaction effect between task and group was not significant (*F*_2,73_ = 0.68, *P* = 0.51, η_p_^2^ = 0.018).

#### P3 Component

The task main effect was significant on P3 amplitude (*F*_1,73_ = 56.68, *P* < 0.001, η_p_^2^ = 0.44), with higher P3 amplitudes during unsuccessful Stop task trials than Go task trials (4.76 ± 0.48 µV vs. 0.77 ± 0.21 µV). There was also a significant interaction between task and group (*F*_2,73_ = 11.94, *P* < 0.001, η_p_^2^ = 0.25). Further simple analysis showed a significant group effect in unsuccessful Stop task trials (*F*_2,73_ = 13.17, *P* < 0.001, η_p_^2^ = 0.27) but not in Go task trials. *Post hoc* analysis revealed reduced P3 amplitude in the SCH group during unsuccessful Stop task trials (6.18 ± 5.74 µV) compared with both the OCD group (10.98 ± 7.15 µV, *P* < 0.05) and HC group (15.06 ± 6.98 µV, *P* < 0.001), while the difference between OCD and HC groups did not reach significance (See detail in **Figure 2**).

#### Event-Related Oscillations

The task effect was significant on the spectral power of theta (*F*_1,73_ = 106.23, *P* < 0.0001, η_p_^2^ = 0.59). Unsuccessful Stop trials elicited stronger power than Go trials (1.71 ± 0.18 dB vs. −0.29 ± 0.12 dB). There was also a significant main effect of electrode cluster (*F*_6,__438_ = 20.10, *P* < 0.001, η_p_^2^ = 0.22), with stronger theta power at middle-frontal areas (1.25 ± 0.14 dB). Moreover, the interaction of task and group was marginally significant (*F*_2,73_ = 3.90, *P* = 0.033, η_p_^2^ = 0.097). The further simple analysis showed that main effect of group was significant for unsuccessful Stop task trials (*F*_2,73_ = 9.32, *P* = 0.017, η_p_^2^ = 0.20). *Post hoc* Bonferroni analysis demonstrated significantly reduced theta power in SCH patients (0.59 ± 0.32) compared with OCD patients (2.15 ± 0.32, *P* = 0.003) and the HC group (2.37 ± 0.31, *P* < 0.001) but no significant difference between OCD and HC groups (See detail in **Figures 3** and **4**).

The task main effect was significant on the ITC (*F*_1,__73_ = 225.09, *P* < 0.001, η_p_^2^ = 0.75), with higher ITC in unsuccessful Stop task trials (0.35 ± 0.01) than in Go task trials (0.18 ± 0.006). Furthermore, the interaction between task and group was significant (*F*_2,73_ = 7.91, *P* = 0.002, η_p_^2^ = 0.18). Further simple analysis showed that the main effect of group was significant in unsuccessful Stop task trials (*F*_2,73_ = 14. 05, *P* < 0.001, η_p_^2^ = 0.28), with significantly lower ITC in the SCH group (0.27 ± 0.02) than in the OCD group (0.35 ± 0.02, *P* = 0.008) and HC group (0.41 ± 0.02, *P* < 0.001). The difference between OCD and HC groups did not reach significance (See detail in **Figures 3** and **4**).

### Correlation Analysis

Pearson correlation analysis revealed marginally significant negative association between Positive Scale PASS score and stop ACC in the SCH group (r = −0.453, *P* = 0.05) (See detail in **Figure 5**). No other significant relationship was found among ERP waves and symptom severity measured by subscale of PANSS and Y-BOCS scores within SCH or OCD group. We also did not found any significant correlations between symptom severity and behavioral performance of SST task within each SCH or OCD group.

## Discussion

The present study directly compared response inhibition among SCH, OCD patients, and healthy age- and sex-matched controls. As expected, both SCH and OCD patients showed impaired response inhibition, as manifested by longer SSRT, reduced P3 amplitudes, weaker theta-band power, and reduced ITC of theta-band activity in successful Stop trials compared to HCs. However, in unsuccessful Stop trials, the SCH group exhibited lower P3 amplitude, theta-band power, and ITC than OCD and HC groups, while the difference in P3 amplitude and theta activity between OCD and HC groups did not reach significance. Collectively, these results suggest similar mechanisms for deficient response inhibition in SCH and OCD patients but mechanistically distinct error processing mechanisms.

As expected, both OCD and SCH group showed slower SSRT compared to HCs. The SSRT provides a temporal window in which to inhibit the initial behavior and is an important index of SST performance. Our findings are consistent with the notion that OCD and SCH patients both require a longer time to stop the prepotent behavior in SSTs, indicating impaired response inhibition (12). In addition, SCH patient demonstrated a significant correlation between PANSS positive symptom subscore and Stop ACC, another important index reflecting the ability for response inhibition. This relationship suggests that decreased response inhibition contributes to the positive symptoms of SCH. Indeed, it has been confirmed that positive symptoms such as auditory hallucinations are linked to dysfunction of the prefrontal cortex, a critical region for response inhibition (32, 33).

In line with previous studies, successful Stop trials elicited larger P3 signals compared to Go trials. The P3 is a classical index of action inhibition processing for successful inhibition of inappropriate responses (8, 34). We found that both SCH and OCD patients exhibited lower P3 amplitudes than HCs in successful Stop task trials, consistent with previous studies (35, 36). These decreased P3 amplitudes indicate that both patient groups have impaired action inhibition. It is well known that like OCD patients, SCH patients demonstrate obsessions, especially during the early onset stage of SCH (37). Lee and colleagues proposed that obsessions predict difficulty in response inhibition (38). The longer SSRT and decreased P3 amplitudes may be linked to obsessions in OCD and SCH groups.

The EROs in the present study also provided important information on the aberrant neural processes underlying deficient response inhibition in SCH and OCD. The temporal location of ERP components and both ERSP and ITC images suggest that the higher P3 amplitude in Stop trials compared to Go trials is most likely generated by theta-band activity. Previous studies have confirmed that theta-band oscillations are critical for cognitive control (39). Under conditions with a low likelihood of a Stop signal (e.g., 30% in this study), participants must inhibit the tendency to respond. The “surprise” Stop signal is coded by increased theta-band activity, which may help to shift the response strategy and to stifle premature Go responses (40). Consistent with ERP results, theta-band power and ITC during successful Stop trials was reduced in OCD and SCH patients within the 200−600-ms time window compared to HCs, providing convergent evidence that altered theta activity may mediate the slower stopping demonstrated by these patients, thereby disrupting efficient inhibitory control (16). This common reduction in theta-band activity has not been reported previously as there are few direct comparisons of EEG activity in OCD and SCH patients performing the same task. Thus, the present study is significant as it implies common neural substrates for impaired response inhibition in these two disorders.

In contrast to successful Stop trials, SCH and OCD groups showed distinct P3 amplitude modulation in unsuccessful Stop trials, with SCH patients exhibiting significantly reduced P3 amplitudes compared to OCD patients and HCs. The neurological significance of P3 elicited in unsuccessful Stop trials is similar to Pe in error processing, and is considered an index of “post-decision” stage processing (7). During this task, the Stop signal provides error feedback to participants. Thus, the P3 could reflect conflict monitoring, conscious error recognition, and response regulation after the Stop signal appears. The error processing indexed by P3 in the present study is related to subjective motivational significance and emotional assessment of error (41, 42). Following this line of interpretation, the smaller P3 amplitude in SCH suggests that errors were of less significance and less distressing compared to OCD patients. Alternatively, OCD patients show overactive performance monitoring and tend to feel dejected about their inappropriate responses and failure to control themselves (43). These results are in line with clinical observations that SCH patients lack self-knowledge, while self-knowledge is intact in OCD.

In addition, we detected increased spectral power and ITC of frontal theta-band activity time locked to unsuccessful Stop trials compared to Go trials at 200−600 ms. A previous study confirmed that the theta-band activity is the neural mechanism of error detection and action regulation, and has similar significance as Pe (11). We found that theta activity, including spectral power and ITC, was also reduced in SCH patients compared to OCD and HC groups. The lower ERN/Pe of SCH patients has been reported in previous studies, but few studies have examined the underlying neural rhythm. It has been proposed that oscillatory electrical activity plays a central role in the recruitment of cerebral systems during information processing (44). The difference between OCD and HC groups did not reach significance, although OCD patients did exhibit weaker activity compared to the HC group. This result is inconsistent with previous studies reporting that OCD patients exhibit stronger theta activity during incorrect responses irrespective of symptom expression (45). This discrepancy may be explained by Riesel et al., who found that the theta oscillation was response-locked. It is unlikely, however, that the theta activity was locked to the stop signal in the present study. In addition, the paradigm used in the study of Riesel et al. was a flanker task. Combined with ERP results, the decreased P3 amplitudes and weaker frontal theta activity in SCH compared to OCD provided evidence that distinct disease-specific neural substrates underlie the unsuccessful stop response. This difference may allow for the objective differentiation of SCH from OCD and facilitate targeted treatment.

The reason we didn’t analyze the response locked EEG data is that in unsuccessful stop task, response locked EEG data could not exclude the neural activity induced by motor execution. It has been confirmed that OCD patients showed higher activity of pre-supplementary area which was important in motor execution (21, 46), which may contaminant the neural signal induced by error processing. In SST, according to horse race model, the unsuccessful stop was on the account that the motor response was sponsored before stop signal appeared(26). Thus, the stop signal could be served as error signal which elicited neural activity of error processing.

Consistent with previous studies, the N2 component was more pronounced in successful and unsuccessful Stop trials than GO trials. N2 is an indicator of automatic conflict monitoring in successful Stop trials or automatic error detection in unsuccessful Stop trials. However, we did not detect any group difference in the N2 component. This suggests that main difference between SCH and OCD is in conscious processing, which is reflected by P3.

Some limitations of this study should be noted. First, almost all SCH patients were taking antipsychotic medication, which may account for some of the differences in neural activity between patient groups. Future studies should investigate unmedicated patients or those with limited antipsychotic drug exposure to confirm our results. Second, it was with great regret that the SCH patient was not assessed by Y-BOCS and OCD patient was not assessed by PANSS to quantify the overlapping comorbidity. However, the overlapping comorbidities were excluded by two professional psychiatrists. For the future research, we will comment the overlapping comorbidity using Third, the number of participants was limited and all were from one region of China. Thus, a larger-scale study enrolling different ethnicities is required to ascertain the relevance of these observations to OCD and SCH patients in general. Finally, EEG has much less precise spatial resolution compared to neuroimaging, so the specific brain regions involved cannot be identified with certainty, such as the frontal-striatum network.

## Conclusion

The behavioral and ERP results demonstrated that both SCH and OCD patients showed impaired response inhibition. However, lower P3 amplitudes and weaker theta activity in SCH patients compared to OCD patients indicated distinct brain activity patterns during error processing in unsuccessful task.

## Data Availability Statement

The datasets generated for this study are available on request to the corresponding author.

## Ethics Statement

The studies involving human participants were reviewed and approved by Ethics committee of Anhui Medical University. The patients/participants provided their written informed consent to participate in this study.

## Author Contributions

FY and XC wrote the first draft of this manuscript and edited the subsequent versions. LZ, TB, and YG are responsible for the data collection and analysis. YD and YL gave critical revision for the manuscript. CZ and KW were responsible for the designing the study. All authors have read and approved the final version of this article.

## Funding

This work was supported by the National Natural Science Foundation of China (91432301, 31771222, 81771456, 31571149, 81171273, and 81803103); and the Natural Science Foundation of Anhui Province (KJ2016A355).

## Conflict of Interest

The authors declare that the research was conducted in the absence of any commercial or financial relationships that could be construed as a potential conflict of interest.

## References

[1] Poyurovsky M Zohar J Glick I Koran LM Weizman R Tandon R Obsessive-compulsive symptoms in schizophrenia: implications for future psychiatric classifications. Compr Psychiatry (2012) 53:480–3. 10.1016/j.comppsych.2011.08.009 22036006

[2] Achim AM Maziade M Raymond E Olivier D Merette C Roy MA How prevalent are anxiety disorders in schizophrenia? A meta-analysis Crit Rev significant assoc. Schizophr Bull (2011) 37:811–21. 10.1093/schbul/sbp148 PMC3122284 19959704

[3] Meier SM Petersen L Pedersen MG Arendt MC Nielsen PR Mattheisen M Obsessive-compulsive disorder as a risk factor for schizophrenia: a nationwide study. JAMA Psychiatry (2014) 71:1215–21. 10.1001/jamapsychiatry.2014.1011 25188738

[4] de Haan L Sterk B Wouters L Linszen DH The 5-year course of obsessive-compulsive symptoms and obsessive-compulsive disorder in first-episode schizophrenia and related disorders. Schizophr Bull (2013) 39:151–60. 10.1093/schbul/sbr077 PMC3523926 21799212

[5] Association AP Schizophrenia spectrum and other psychotic disorders. In: DISORDERS D. A. S. M. O. M. editor. Eur Child Adolesc Psychiatry.American Psychiatric Association (2013). 10.1007/s00787-012-0354-x 23202885

[6] Ethridge LE Soilleux M Nakonezny PA Reilly JL Hill SK Keefe RS Behavioral response inhibition in psychotic disorders: diagnostic specificity, familiality and relation to generalized cognitive deficit. Schizophr Res (2014) 159:491–8. 10.1016/j.schres.2014.08.025 PMC4253557 25261042

[7] Hoptman MJ Parker EM Nair-Collins S Dias EC Ross ME Dicostanzo JN Sensory and cross-network contributions to response inhibition in patients with schizophrenia. NeuroImage Clin (2018) 18:31–9. 10.1016/j.nicl.2018.01.001 PMC5984577 29868440

[8] Kok A Ramautar JR Ruiter MB Band GP Ridderinkhof KR ERP components associated with successful and unsuccessful stopping in a stop-signal task. Psychophysiology (2004) 41:9–20. 10.1046/j.1469-8986.2003.00127.x 14692996

[9] Dippel G Chmielewski W Muckschel M Beste C Response mode-dependent differences in neurofunctional networks during response inhibition: an EEG-beamforming study. Brain Struct Funct (2016) 221:4091–101. 10.1007/s00429-015-1148-y 26608829

[10] de Vega M Morera Y Leon I Beltran D Casado P Martin-Loeches M Sentential negation might share neurophysiological mechanisms with action inhibition. Evidence from frontal theta rhythm. J Neurosci (2016) 36:6002–10. 10.1523/JNEUROSCI.3736-15.2016 PMC6601810 27251621

[11] Trujillo LT Allen JJ Theta EEG dynamics of the error-related negativity. Clin Neurophysiol. (2007) 118:645–68. 10.1016/j.clinph.2006.11.009 17223380

[12] Hughes ME Fulham WR Johnston PJ Michie PT Stop-signal response inhibition in schizophrenia: behavioural, event-related potential and functional neuroimaging data. Biol Psychol (2012) 89:220–31. 10.1016/j.biopsycho.2011.10.013 22027085

[13] Patel A Everitt B Knapp M Reeder C Grant D Ecker C Schizophrenia patients with cognitive deficits: factors associated with costs. Schizophr Bull (2006) 32:776–85. 10.1093/schbul/sbl013 PMC2632261 16885205

[14] Thakkar KN Schall JD Boucher L Logan GD Park S Response inhibition and response monitoring in a saccadic countermanding task in schizophrenia. Biol Psychiatry (2011) 69:55–62. 10.1016/j.biopsych.2010.08.016 20970778 PMC3006077

[15] Mayer AR Hanlon FM Dodd AB Yeo RA Haaland KY Ling JM Proactive response inhibition abnormalities in the sensorimotor cortex of patients with schizophrenia. J Psychiatry Neurosci (2016) 41:312–21. 10.1503/jpn.150097 PMC5008920 26883319

[16] Cooper PS Hughes ME Impaired theta and alpha oscillations underlying stopsignal response inhibition deficits in schizophrenia. Schizophr Res (2018) 193:474–6. 10.1016/j.schres.2017.08.002 28797527

[17] Chamberlain SR Fineberg NA Blackwell AD Robbins TW Sahakian BJ Motor inhibition and cognitive flexibility in obsessive-compulsive disorder and trichotillomania. Am J Psychiatry (2006) 163:1282–4. 10.1176/ajp.2006.163.7.1282 16816237

[18] Penades R Catalan R Rubia K Andres S Salamero M Gasto C Impaired response inhibition in obsessive compulsive disorder. Eur Psychiatry (2007) 22:404–10. 10.1016/j.eurpsy.2006.05.001 17127038

[19] Lipszyc J Schachar R Inhibitory control and psychopathology: a meta-analysis of studies using the stop signal task. J Int Neuropsychol. Soc (2010) 16:1064–76. 10.1017/S1355617710000895 20719043

[20] Lei H Zhu X Fan J Dong J Zhou C Zhang X Is impaired response inhibition independent of symptom dimensions in obsessive-compulsive disorder? Evidence ERPs. Sci Rep (2015) 5:10413. 10.1038/srep10413 25990063 PMC4438428

[21] de Wit SJ De Vries FE Van Der Werf YD Cath DC Heslenfeld DJ Veltman EM Presupplementary motor area hyperactivity during response inhibition: a candidate endophenotype of obsessive-compulsive disorder. Am J Psychiatry (2012) 169:1100–8. 10.1176/appi.ajp.2012.12010073 23032388

[22] Endrass T Ullsperger M Specificity of performance monitoring changes in obsessive-compulsive disorder. Neurosci Biobehav Rev (2014) 46 Pt 1:124–38. 10.1016/j.neubiorev.2014.03.024 24747486

[23] Hanna GL Liu Y Isaacs YE Ayoub AM Brosius A Salander Z Error-related brain activity in adolescents with obsessive-compulsive disorder and major depressive disorder. Depress Anxiety (2018) 35:752–60. 10.1002/da.22767 PMC6105517 29734494

[24] Foti D Kotov R Bromet E Hajcak G Beyond the broken error-related negativity: functional and diagnostic correlates of error processing in psychosis. Biol Psychiatry (2012) 71:864–72. 10.1016/j.biopsych.2012.01.007 PMC3334442 22336564

[25] Goodman WK Price LH Rasmussen SA Mazure C Fleischmann RL Hill CL The Yale-Brown Obsessive Compulsive Scale. I. Development, use, and reliability. Arch Gen Psychiatry (1989) 46:1006–11. 10.1001/archpsyc.1989.01810110048007 2684084

[26] Verbruggen F Logan GD Models of response inhibition in the stop-signal and stop-change paradigms. Neurosci Biobehav Rev (2009) 33:647–61. 10.1016/j.neubiorev.2008.08.014 PMC2696813 18822313

[27] Delorme A Makeig S EEGLAB: an open source toolbox for analysis of single-trial EEG dynamics including independent component analysis. J Neurosci Methods (2004) 134:9–21. 10.1016/j.jneumeth.2003.10.009 15102499

[28] Delorme A Palmer J Onton J Oostenveld R Makeig S Independent EEG sources are dipolar. PloS One (2012) 7:e30135. 10.1371/journal.pone.0030135 22355308 PMC3280242

[29] Chaumon M Bishop DV Busch NA A practical guide to the selection of independent components of the electroencephalogram for artifact correction. J Neurosci Methods (2015) 250:47–63. 10.1016/j.jneumeth.2015.02.025 25791012

[30] Klipper-Aurbach Y Wasserman M Braunspiegel-Weintrob N Borstein D Peleg S ASSA S Mathematical formulae for the prediction of the residual beta cell function during the first two years of disease in children and adolescents with insulin-dependent diabetes mellitus. Med Hypotheses (1995) 45:486–90. 10.1016/0306-9877(95)90228-7 8748093

[31] Cohen J Eta-squared and partial eta-squared in fixed factor ANOVA designs. Educ Psychol Meas. (1973) 33:107–12. 10.1177/001316447303300111

[32] Bose A Shivakumar V Narayanaswamy JC Nawani H Subramaniam A Agarwal SM Insight facilitation with add-on tDCS in schizophrenia. Schizophr Res (2014) 156:63–5. 10.1016/j.schres.2014.03.029 24767881

[33] Chen X Ji GJ Zhu C Bai X Wang L He K Neural Correlates of Auditory Verbal Hallucinations in Schizophrenia and the Therapeutic Response to Theta-Burst Transcranial Magnetic Stimulation. Schizophr Bull (2018) 45(2):474–83. 10.1093/schbul/sby054 PMC6403092 29733409

[34] Ramautar JR Kok A Ridderinkhof KR Effects of stop-signal modality on the N2/P3 complex elicited in the stop-signal paradigm. Biol Psychol (2006) 72:96–109. 10.1016/j.biopsycho.2005.08.001 16157441

[35] Morein-Zamir S Fineberg NA Robbins TW Sahakian BJ Inhibition of thoughts and actions in obsessive-compulsive disorder: extending the endophenotype? Psychol Med (2010) 40(2):263–72. 10.1017/S003329170999033X PMC2829968 19573261

[36] Krakowski MI Sanctis P Foxe JJ Hoptman MJ Nolan K Kamiel S Disturbances in response inhibition and emotional processing as potential pathways to violence in schizophrenia: a high-density event-related potential study. Schizophr Bull (2016) 42:963–74. 10.1093/schbul/sbw005 PMC4903062 26895845

[37] Swets M Dekker J Van Emmerik-Van Oortmerssen K Smid GE Smit F De Haan L The obsessive compulsive spectrum in schizophrenia, a meta-analysis and meta-regression exploring prevalence rates. Schizophr Res (2014) 152:458–68. 10.1016/j.schres.2013.10.033 24361303

[38] Lee HJ Yost BP Telch MJ Differential performance on the go/no-go task as a function of the autogenous-reactive taxonomy of obsessions: findings from a non-treatment seeking sample. Behav Res Ther (2009) 47:294–300. 10.1016/j.brat.2009.01.002 19217612

[39] Cavanagh JF Frank MJ Frontal theta as a mechanism for cognitive control. Trends Cognit Sci (2014) 18:414–21. 10.1016/j.tics.2014.04.012 PMC4112145 24835663

[40] Dippel G Muckschel M Ziemssen T Beste C Demands on response inhibition processes determine modulations of theta band activity in superior frontal areas and correlations with pupillometry - Implications for the norepinephrine system during inhibitory control. Neuroimage (2017) 157:575–85. 10.1016/j.neuroimage.2017.06.037 28647483

[41] Hajcak G Foti D Errors are aversive: defensive motivation and the error-related negativity. Psychol Sci (2008) 19:103–8. 10.1111/j.1467-9280.2008.02053.x 18271855

[42] Wu J Sun X Wang L Zhang L Fernandez G Yao Z Error consciousness predicts physiological response to an acute psychosocial stressor in men. Psychoneuroendocrinology (2017) 83:84–90. 10.1016/j.psyneuen.2017.05.029 28601751

[43] Riesel A Endrass T Auerbach LA Kathmann N Overactive Performance Monitoring as an endophenotype for obsessive-compulsive disorder: evidence from a treatment study. Am J Psychiatry (2015) 172:665–73. 10.1176/appi.ajp.2014.14070886 25783756

[44] Lakatos P Karmos G Mehta AD Ulbert I Schroeder CE Entrainment of neuronal oscillations as a mechanism of attentional selection. Science (2008) 320:110–3. 10.1126/science.1154735 18388295

[45] Riesel A Kathmann N Endrass T Overactive performance monitoring in obsessive-compulsive disorder is independent of symptom expression. Eur Arch Psychiatry Clin Neurosci (2014) 264:707–17. 10.1007/s00406-014-0499-3 24676800

[46] Nachev P Kennard C Husain M Functional role of the supplementary and pre-supplementary motor areas. Nat Rev Neurosci (2008) 9:856–69. 10.1038/nrn2478 18843271

